# The Role of the Histone Methyltransferase PfSET10 in Antigenic Variation by Malaria Parasites: a Cautionary Tale

**DOI:** 10.1128/mSphere.01217-20

**Published:** 2021-02-03

**Authors:** Che J. Ngwa, Mackensie R. Gross, Jean-Pierre Musabyimana, Gabriele Pradel, Kirk W. Deitsch

**Affiliations:** a Division of Cellular and Applied Infection Biology, Institute of Zoology, RWTH Aachen University, Aachen, Germany; b Department of Microbiology and Immunology, Weill Cornell Medical College, New York, New York, USA; University of Copenhagen

**Keywords:** histone methyltransferase, epigenetic gene regulation, malaria, antigenic variation, chromatin modifications

## Abstract

The virulence of the malaria parasite Plasmodium falciparum is due in large part to its ability to avoid immune destruction through antigenic variation. This results from changes in expression within the multicopy *var* gene family that encodes the surface antigen P. falciparum erythrocyte protein one (PfEMP1). Understanding the mechanisms underlying this process has been a high-profile research focus for many years. The histone methyltransferase PfSET10 was previously identified as a key enzyme required both for parasite viability and for regulating *var* gene expression, thus making it a prominent target for developing antimalarial intervention strategies and the subject of considerable research focus. Here, however, we show that disruption of the gene encoding PfSET10 is not lethal and has no effect on *var* gene expression, in sharp contrast with previously published reports. The contradictory findings highlight the importance of reevaluating previous conclusions when new technologies become available and suggest the possibility of a previously unappreciated plasticity in epigenetic gene regulation in P. falciparum.

**IMPORTANCE** The identification of specific epigenetic regulatory proteins in infectious organisms has become a high-profile research topic and a focus for several drug development initiatives. However, studies that define specific roles for different epigenetic modifiers occasionally report differing results, and we similarly provide evidence regarding the histone methyltransferase PfSET10 that is in stark contrast with previously published results. We believe that the conflicting results, rather than suggesting erroneous conclusions, instead reflect the importance of revisiting previous conclusions using newly developed methodologies, as well as caution in interpreting seemingly contrary results in fields that are known to display considerable plasticity, for example metabolism and epigenetics.

## Abstract (precis)

The identification of specific epigenetic regulatory proteins in infectious organisms has become a high-profile research topic and a focus for several drug development initiatives. However, studies that define specific roles for different epigenetic modifiers occasionally report differing results, and we similarly provide evidence regarding the histone methyltransferase PfSET10 that is in stark contrast with previously published results.

## OBSERVATION

A major contributor to the pathogenesis of Plasmodium falciparum, the most virulent human malaria parasite, is the propensity of infected red blood cells (RBCs) to cytoadhere to the vascular endothelium, leading to localized inflammation and tissue damage (1). This property results from the placement of the variant adhesive receptor P. falciparum erythrocyte protein one (PfEMP1) onto the infected cell surface. Different forms of PfEMP1 are encoded by members of the multicopy *var* gene family, and switches in which gene is expressed alter the PfEMP1 variant that is displayed. This process, called antigenic variation, enables the parasites to avoid the antibody response of the host. Thus, the virulence of P. falciparum infections and their chronic nature are inherently linked to *var* gene expression, making this a high-profile research topic that has gained considerable attention.

Expression of *var* genes is regulated epigenetically through the deposition of specific histone marks at active and silent genes and through changes in subnuclear localization (reviewed in reference 2). Specifically, silent genes are clustered within regions of condensed chromatin at the nuclear periphery, while the active gene is located within a specific, euchromatic “expression site” associated with active transcription (3, 4). In seminal work from Volz and colleagues, these two important aspects were linked with the report that the H3K4 methyltransferase PfSET10 was uniquely localized to the *var* expression site. The authors further provided evidence that PfSET10 was required to maintain the active *var* gene in a poised state during cellular division, thus enabling reactivation of the gene in daughter parasites (5). This evidence implicated PfSET10 as required for the maintenance of epigenetic memory, a property essential for antigenic variation. More importantly, the paper provided a new conceptual framework for how subnuclear localization could contribute to epigenetic gene regulation in malaria parasites and identified PfSET10 as a key contributor to *var* gene regulation and therefore as a potential target for the development of new disease intervention strategies.

While the evidence provided by Volz et al. was compelling, the authors were unable to knock out the *Pfset10* gene and thus could not definitively demonstrate its necessity for the maintenance of *var* epigenetic memory. They therefore proposed that PfSET10 has an additional, vital function, since *var* gene expression is not required for viability in culture. It was therefore surprising that a recent genome-wide transposon mutagenesis screen in P. falciparum identified eight independent insertions within the *Pfset10* coding region, each expected to disrupt gene function, thus indicating that the gene is dispensable for parasite viability (6). The contradictory results of these two high-profile studies raise questions about our current understanding of epigenetic gene regulation in malaria parasites and the best direction for future studies in this field. We therefore aimed to address this discrepancy through targeted gene disruption of the *Pfset10* locus in the same genetic background of P. falciparum, 3D7, as that originally used by Volz et al.

**FIG 1:**
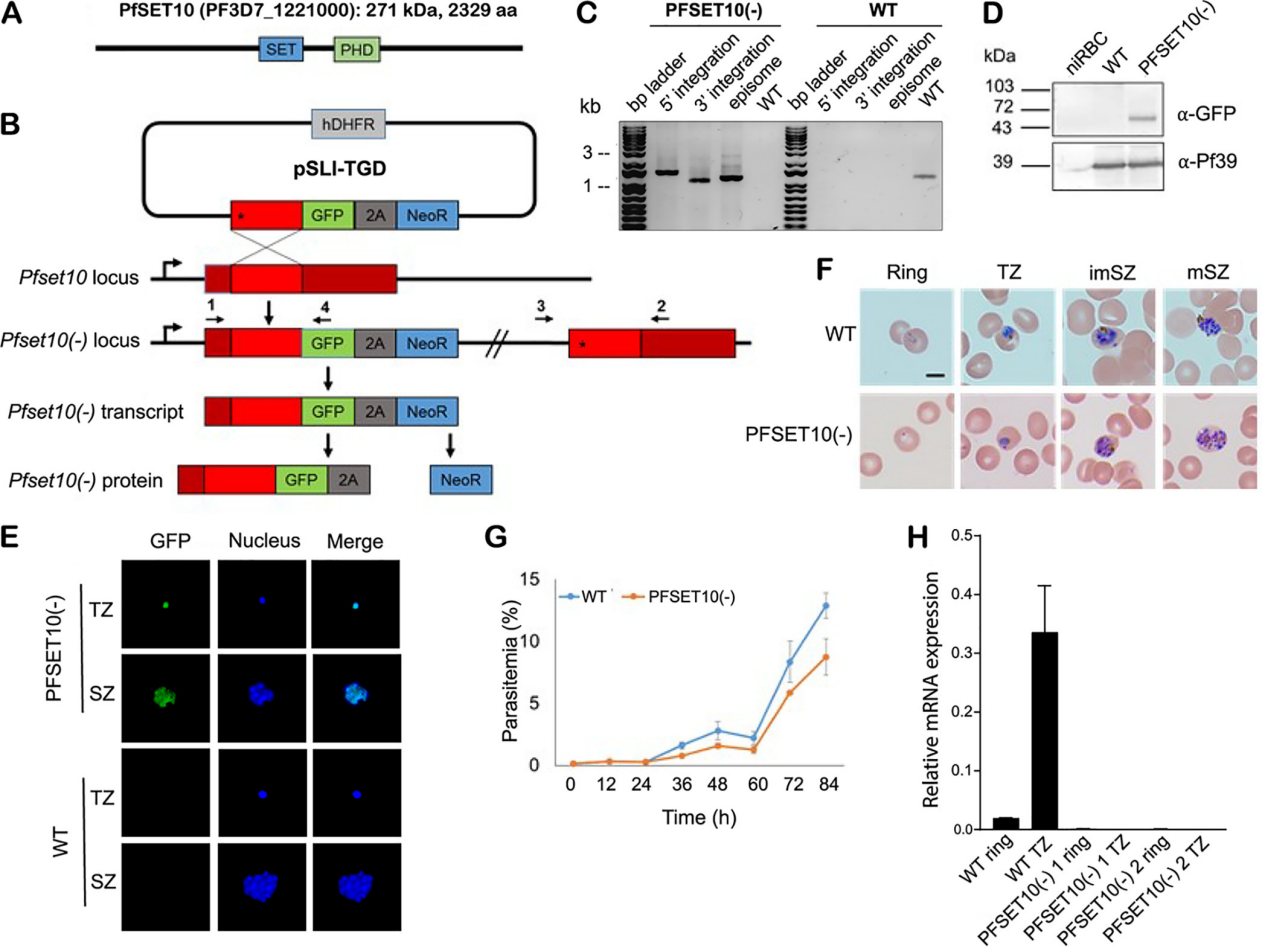
Analysis of PfSET10(−) asexual blood-stage parasites. (A) Schematic depicting PfSET10. The SET (blue box) and PHD zinc finger (green box) domains are indicated. aa, amino acid. (B) Schematic depicting the gene knockout (KO) strategy via single crossover recombination-based gene disruption using selection-linked integration-mediated targeted gene disruption (SLI-TGD). The vector pSLI-TGD was modified to contain a 900-bp sequence block (light red box) from near the 5′ end of the *Pfset10* coding region (dark red box). The coding region was maintained in frame with a green fluorescent protein coding region (green box), a 2A “skip” peptide (gray box), and the Neo-R gene (blue) that provides resistance to the antibiotic G418. Medium containing G418 selects for integration into the locus and disruption of the *Pfset10* coding region. Arrows indicate the position of primers 1 to 4 used to detect integration of the pSLI-TGD vector. Asterisks indicate a stop codon. GFP, green fluorescent protein; hDHFR; human dihydrofolate reductase for resistance to WR99210; NeoR, neomycin resistance; 2A, skip peptide. (C) Confirmation of vector integration for the PfSET10(−) parasites by diagnostic PCR using genomic DNA (gDNA) obtained from PfSET10(−) and the wild type (WT; P. falciparum strain NF54). 5′ Integration was detected using primers 1 and 4 (1,470 bp), and 3′ integration was detected using primers 2 and 3 (1,164 bp). Primers 3 and 4 were used to detect the presence of episomes (1,251 bp), and primers 1 and 2 were used for WT control (1,342 bp). (D) Confirmation of truncated PfSET10 tagged with GFP. Parasite lysates were subjected to Western blotting using polyclonal mouse anti-GFP (67 kDa). Lysates of WT and noninfected red blood cells (niRBC) were used as negative controls. Immunoblotting with mouse anti-Pf39 antiserum (39 kDa) served as a loading control. (E) Verification of GFP expression in the PfSET10(−) parasites. Live images of trophozoites (TZ) and schizonts (SZ) of the PfSET10(−) line detected GFP (green) associated with the parasite nuclei. The WT was used for a negative control. Nuclei were counterstained by Hoechst 33342 (blue). Bar, 5 μm. (F) Morphology of the PfSET10(−) asexual blood-stages. The morphology was compared via Giemsa staining of asexual blood stages of PfSET10(−) and the WT. TR, trophozoite; imSZ, immature schizont; mSZ, mature schizont. Bar, 5 μm. (G) Asexual blood stage replication of the PfSET10(−) line. Synchronized ring stage cultures of WT and PfSET10(−) with a starting parasitemia of 0.25% were maintained in cell culture medium, and the parasitemia was followed via Giemsa smears over a time period of 0 to 84 h. The experiment was performed in triplicate (mean ± standard deviation [SD]). (H) Steady-state *Pfset10* mRNA levels of WT and two PfSET10(−) lines. qRT-PCR was used to detect expression levels in both rings and trophozoite-stage parasites. Expression levels are displayed relative to *seryl-tRNA ligase*. Results shown in panels C to H are representative of two to three independent biological replicates.

PfSET10 is a 271-kDa protein that comprises a central SET domain and a PHD zinc finger domain (Fig. 1A). We utilized selection-linked integration-mediated targeted gene disruption (SLI-TGD) (7) to directly disrupt *Pfset10* (Fig. 1B and Text S1). We were readily able to obtain parasites in which the targeting construct was integrated into the coding region of the gene (Fig. 1C) and which displayed neomycin resistance. The transgenic parasites expressed a truncated N-terminal fragment of PfSET10; however, expression of both the SET and PHD domains was eliminated, rendering the line an enzymatic knockout. We therefore refer to this line as PfSET10(−). The remainder of the protein is fused to green fluorescent protein (GFP), which could be detected by Western blotting and live imaging (Fig. 1D and E). The asexual blood-stage parasites displayed normal morphologies (Fig. 1F) and exhibited only slightly reduced intraerythrocytic growth compared to the wild type (WT), with normal progression through the replicative cycle (Fig. 1G), consistent with the gene being nonessential for viability.

**FIG 2:**
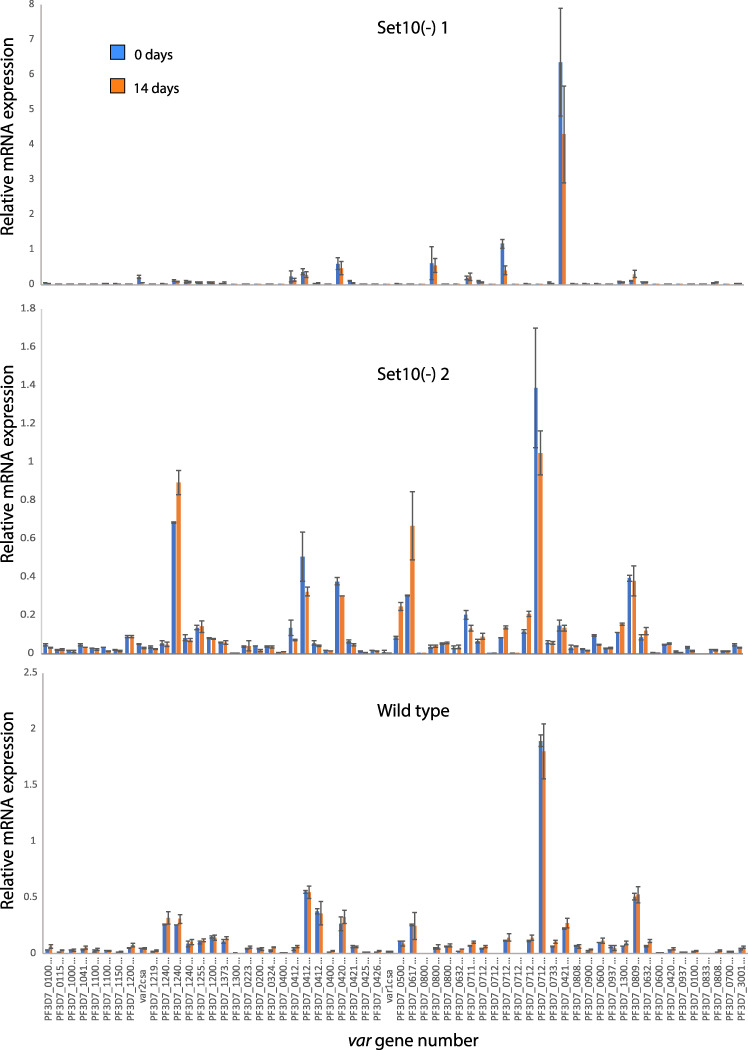
Assessment of *var* gene expression in WT and two PfSET10(−) lines. Steady-state mRNA levels for each *var* gene were determined using qRT-PCR and displayed relative to expression of *seryl-tRNA ligase*. RNA was extracted from each line at an initial time point (0 days, blue) and after 2 weeks of continuous culture (14 days, orange). Expression profiles for each knockout line (top and middle panels), as well as for wild-type parasites (bottom panel), are shown. The annotation number for each *var* gene is shown on the *x* axis of the bottom panel. Results are representative of three independent experiments.

Quantitative reverse transcriptase PCR (qRT-PCR) analysis of gene expression failed to detect intact *Pfset10* transcripts in two parasite lines used for transcript analyses (Fig. 1H), confirming disruption of the *Pfset10* gene. To assess *var* gene expression, RNA was extracted from synchronized cultures of both WT and PfSET10(−) parasites ∼16 h after RBC invasion when *var* mRNA levels peak. Transcript levels for each individual *var* gene were assessed using a standardized qRT-PCR assay (8). These experiments detected similar patterns of *var* mRNA expression in the WT and the PfSET10(−) lines, indicating that the methyltransferase activity of PfSET10 is not required for *var* gene expression (Fig. 2). Furthermore, assays of parasites grown in continuous culture for an additional 2 weeks (7 generations) detected nearly identical expression patterns, indicating only minimal *var* expression switching and thereby demonstrating that epigenetic memory remained intact in the PfSET10(−) lines (Fig. 2). If PfSET10 was required for the maintenance of epigenetic memory and to preserve the poised state of the active *var* gene, as concluded by Volz et al., the knockout lines would be expected to display either no *var* gene expression or extremely accelerated switching leading to expression of the entire gene family within the parasite population. In contrast, we detected no discernible effect on *var* gene expression in these lines.

The results described here are in stark contrast with the original conclusions of Volz et al., who concluded that PfSET10 is required for both *var* gene regulation and parasite viability (5). These authors were unable to obtain viable PfSET10 knockout lines despite applying three separate approaches, which were state of the art at that time. It is possible that by using SLI-TGD, a method that enables strong selection pressure to obtain the targeted integration, we were able to obtain a *Pfset10* disruption in a way that was not previously possible. However, it is worth noting that the saturation mutagenesis study of Zhang et al. did not employ strong selection for targeted integration but nonetheless readily obtained multiple, independent *Pfset10* disruptions, suggesting that selection pressure alone is not responsible for the differing results. An alternative explanation is that the parasites in our study and those used for the saturation mutagenesis study of Zhang et al. (6) were able to compensate for the loss of PfSET10 through alteration of other epigenetic pathways. For example, the P. falciparum genome encodes three additional proteins predicted to have H3K4 methyltransferase activity, *Pfset1*, *Pfset4*, and *Pfset6*, and modified activity of one of these alternative histone methyltransferases could potentially lessen or eliminate the detrimental effects of the loss of the methyltransferase activity of PfSET10. Plasticity of epigenetic pathways that control gene expression has been observed in mammalian systems (9); for example, in human cells the H3K27 methyltransferases EZH1 and EZH2 have been shown to compensate for one another when the activity of one protein is lost (10, 11).

If such plasticity is a common aspect of epigenetic gene regulation in malaria parasites, this could explain other contradictory results previously reported regarding the epigenetic control of gene regulation in P. falciparum. For example, disruption of the histone deacetylase genes *Pfsir2a* and *Pfsir2b* were originally reported to cause profound changes in *var* gene expression (12, 13), while a subsequent study observed little to no effect of *Pfsir2b* disruption in some lines (14). Investigations into the roles of RecQ helicases in *var* gene regulation have been similarly contradictory, with one study reporting that knockout of either *PfRecQ1* or *PfWRN* caused dysregulation of large subsets of the *var* gene family (15) and a second study showing that disruption of *PfWRN* had no effect on *var* gene expression, whereas knocking out *PfRecQ1* silenced the entire *var* gene family (16). Several scenarios can easily be imagined that could provide an explanation for these contrary observations. For example, changes in enzymatic activity, subnuclear localization, or recruitment to alternative genomic loci of any protein involved in epigenetic regulation could partially or fully compensate for loss of an experimentally targeted epigenetic regulator, thereby resulting in very different phenotypes in different parasite lines despite similar or identical genetic modifications. Considerable caution should therefore be exercised when interpreting the results of such experiments. Inhibitors of epigenetic enzymes are actively being explored as potential new antimalarial drugs; however, the potential for parasites to compensate will need to be carefully considered to avoid rapid development of drug resistance.

10.1128/mSphere.01217-20.1 TEXT S1 Experimental procedures. Detailed information describing the following experimental procedures are described here: parasite culture, generation of PfSET10(−) parasites, analysis of *PfSet10* and *var* steady-state RNA, live imaging and microscopy, asexual blood-stage replication assay, and Western blotting. Download Text S1, DOCX file, 0.03 MB. Copyright © 2021 Ngwa et al. 2021 Ngwa et al. This content is distributed under the terms of the Creative Commons Attribution 4.0 International license.
