# Supplementary material for: The Role of the Histone Methyltransferase PfSET10 in Antigenic Variation by Malaria Parasites: a Cautionary Tale
Source: mSphere. 2021 Feb 3;6(1):e01217-20. doi: 10.1128/mSphere.01217-20 (PMC7860991; doi:10.1128/mSphere.01217-20)
Supplement: TEXT S1 [file mSphere.01217-20-s0001.docx]

**Supplemental Material**

EXPERIMENTAL PROCEDURES

Parasite culture - The *P. falciparum* strain NF54 was cultivated in vitro in RPMI 1640 medium (Gibco). The medium was initially supplemented with 10% v/v heat-inactivated human serum (HIS) and A+ erythrocytes at a 2% v/v hematocrit as described (1, 2). For Q-PCR analyses, parasites were cultured in media containing 0.5% Albumax II (Life Technologies), 0.25% sodium bicarbonate and 0.1 mg/ml gentamicin. PfSET10(-) parasites were cultured in media containing 400 μg/ml G418 (Sigma-Aldrich). The parasites were incubated at 37°C in an atmosphere of 5% oxygen, 5% carbon dioxide, and 90% nitrogen. Human serum and erythrocyte concentrate were obtained from the Department of Transfusion Medicine, University Hospital Aachen, Germany. Donor sera and blood samples were pooled and kept anonymous. The work with human blood was approved by the Ethics commission of the RWTH University Hospital.

Generation of PfSET10(-) parasites - Disruption of *Pfset10* (Pf3D7_1221000) was obtained by selection-linked integration as described by Birnbaum et al (3). Briefly, the plasmid pSLI-TGD-GFP was modified to contain a 900 bp homology block from the 5’ end of the Pfset10 coding region. Parasites were transfected as described (4) and stably transformed lines maintained through selection with 4 nm WR99210. To select for integration and disruption of *Pfset10*, parasites were cultured in media containing 400 μg/ml G418. Parasites resistant to G418 were cloned and correct integration confirmed by PCR using primers specific to both the 5’ and 3’ ends of the integrated construct as shown in Figure 1B. Primer sequences: primer 1 = 5’-GAGTGTCAGTTTTTATGTTTTCTT-3’; primer 2 = 5’-CAAGTGTTGGCCATGGAA-3’; primer 3= GCTTTACACTTTATGCTTCCGGCTCG-3’; primer 4 = 5’- TTTCTTTTTGTGGGTTCGGT-3’. The PCR conditions used were: annealing for 15 seconds at 50^o^ C for detection of 3’ integration, episomes and WT locus or at 54^o^ C for detection of 5’ integration; extension at 60^o^ C for 2 minutes for all reactions; 35 cycles.

Analysis of *PfSet10* and *var* steady state RNA – Parasites were synchronized using sorbitol lysis (5) and RNA was extracted from synchronized ring or late trophozoite stage parasites. RBCs were lysed using 10% saponin solution and RNA extraction was performed using Trizol LS Reagent (Invitrogen) as described (6). RNA was purified using the PureLink RNA Mini Kit (Ambion) followed by treatment with DNase I (Invitrogen). cDNA synthesis was performed with Superscript II RNase H reverse transcriptase (Invitrogen) with random primers (Invitrogen) as described by the manufacturer. 800 ng to 1 μg of total RNA was used for each cDNA synthesis reaction and a control reaction without reverse transcriptase was performed in parallel. Quantitative Reverse Transcriptase PCR (qRT-PCR) analysis was done using the relative standard curve method (Applied Biosystems) using *seryl-tRNA ligase* (PF3D7_0717700) for normalization. To ensure reproducibility, all assays were repeated using the alternative genes *fructose biphosphate aldolase* (PF3D7_1444800) and *actin* (PF3D7_1246200) for normalization. All qRT-PCR reactions were performed in duplicate with a QuantStudio 6 system (Thermofischer) using iTaq SYBR Green Supermix (Bio-Rad). For *Pfset10*, the primers pair 5’–GGATCATCCCAATAAAGCTTTAC–3’ and 5’–TTGTTGTTTTCACAGAATTTCC–3’ were used. For *var* gene expression, a standardized primer set that detects transcripts from the entire *var* gene family (7) with modifications described by (8) was used.

Live Imaging and microscopy – Mixed asexual blood stage cultures of WT and PfSET10(-) parasites were incubated with Hoechst 33342 nuclear stain for 10 min at room temperature (RT) to highlight the nuclei. The cells were seeded as cell monolayers on glass slides and imaged using a Leica DM 5500 B microscope. Digital images were processed using the Adobe Photoshop CS software.

Asexual blood stage replication assay –To compare the asexual blood stage replication between WT and PfSET10(-) parasites, synchronized asexual blood stage cultures were set to an initial parasitemia of 0.25% ring stages und cultivated as described above. Giemsa-stained thin blood smears were prepared every 12 h over a time span of 84 h. The parasitemia of each time point was determined microscopically at 1,000-fold magnification by counting the percentage of parasites in 1,000 red blood cells.

Western Blotting – Asexual blood stages of WT and PfSET10(-) parasites were harvested from cultures. For erythrocytes lysis, parasites were incubated with 0.05% w/v saponin/PBS for 10 min on ice. Parasites were pelleted and then resuspended in lysis buffer (0.5% Triton X-100, 4% w/v SDS, 0.5xPBS) supplemented with protease inhibitor cocktail (complete EDTA-free; Roche Diagnostics Germany). Non-infected red blood cell lysates were used as negative control. 5xSDS-PAGE loading buffer containing 25 mM dithiothreitol was added to the lysates, samples were heat-denatured for 10 min at 95°C and separated via SDS-PAGE. Following gel electrophoresis, separated parasite proteins were transferred to Hybond ECL nitrocellulose membrane (Amersham Biosciences) according to the manufacturer’s protocol. Non-specific binding was blocked by incubation of the membranes in Tris-buffered saline containing 5% w/v skim milk, pH 7.5, for 1 h at RT. For immunodetection, membranes were incubated with polyclonal mouse anti-GFP antisera (dilution 1:700; Roche) or polyclonal rabbit anti-Pf39 antisera (dilution 1:10,000) for 2 h at RT. After washing, membranes were incubated with the respective alkaline phosphatase-conjugated secondary antibody (Sigma-Aldrich) for 1 h at RT and developed in a solution of nitroblue tetrazolium chloride (NBT) and 5-brom-4-chlor-3-indoxylphosphate (BCIP; Sigma-Aldrich) for 5-30 min at RT. Blots were scanned and processed using the Adobe Photoshop CS software.

**SM References**

1. W. Trager, J. B. Jensen, Human malaria parasites in continuous culture. *Science* **193**, 673-675 (1976).

2. T. Ifediba, J. P. Vanderberg, Complete in vitro maturation of Plasmodium falciparum gametocytes. *Nature* **294**, 364-366 (1981).

3. J. Birnbaum *et al.*, A genetic system to study Plasmodium falciparum protein function. *Nat Methods* **14**, 450-456 (2017).

4. Y. Wu, C. D. Sifri, H.-H. Lei, X. Su, T. E. Wellems, Transfection of *Plasmodium falciparum* within human red blood cells. *Proceedings of the National Academy of Sciences USA* **92**, 973-977 (1995).

5. C. Lambros, J. P. Vanderberg, Synchronization of Plasmodium falciparum erythrocytic stages in culture. *J. Parasitol* **65**, 418-420 (1979).

6. S. Kyes, R. Pinches, C. Newbold, A simple RNA analysis method shows var and rif multigene family expression patterns in Plasmodium falciparum. *Mol. Biochem. Parasitol* **105**, 311-315 (2000).

7. A. Salanti *et al.*, Selective upregulation of a single distinctly structured var gene in chondroitin sulphate A-adhering Plasmodium falciparum involved in pregnancy-associated malaria. *Molecular Microbiology* **49**, 179-191 (2003).

8. M. Frank *et al.*, Strict pairing of var promoters and introns is required for var gene silencing in the malaria parasite plasmodium falciparum. *J. Biol. Chem* **281**, 9942-9952 (2006).
